# Supplementary material for: Interprofessional collaboration and associated factors among wound, ostomy, and continence nurses: a cross-sectional study in China
Source: PeerJ. 2025 Sep 30;13:e20006. doi: 10.7717/peerj.20006 (PMC12493770; doi:10.7717/peerj.20006)
Supplement: Supplemental Information 4 [file peerj-13-20006-s004.docx]

****Survey on inter-professional collaboration competency among** wound ostomy continence **nurses****

**Part 1**

**Demographic Information**

1.Gender:
○ male　○ female

2.Age (in years): ___________________

3.Are you an only child?
○ yes
○ no

4.Your marital status:
○ unmarried
○ married

5.Your specialty working years:___________________

6.Your professional title:
○ primary

○ intermediate

○ senior

7.Your administrative position:
○ no
○ nursing manager

8.Highest educational level:
○ associate degree
○ bachelor’s degree
○ master’s degree

○ doctor's degree

9.Hospital level:
○ tertiary hospital

○ secondary hospital

○ primary hospital

10.Do you have Off-site learning experience in the past three years?
○ no
○ yes

11.Your employment type:
○ authorized
○ contract

12.Your work pattern:

○ full-time
○ part-time

13.Do you serve as a specialty team leader?
○ yes
○ no

****Part 2** Chiba inter-professional competency scale (CICS29)**

****When you collaborate with other professionals, how do you understand and implement collaboration? Please select the appropriate option from the following items. The "team" in this scale refers to a team composed of multiple professionals for the purpose of patient treatment and care (such as a team composed of wound ostomy continence nurses nurses and doctors from different disciplines and other professionals).****

1.I constantly strive to improve my performance

| ○strongly agree |
| --- |
| ○agree |
| ○neutral |
| ○disagree |
| ○strongly disagree |

2.I always reflect on the care that I have provided

| ○strongly agree |
| --- |
| ○agree |
| ○neutral |
| ○disagree |
| ○strongly disagree |

3.I strive to be a professional

| ○strongly agree |
| --- |
| ○agree |
| ○neutral |
| ○disagree |
| ○strongly disagree |

4.I practice evidence-based care

| ○strongly agree |
| --- |
| ○agree |
| ○neutral |
| ○disagree |
| ○strongly disagree |

5.I am able to explain the basis for care to anyone

| ○strongly agree |
| --- |
| ○agree |
| ○neutral |
| ○disagree |
| ○strongly disagree |

6.I am able to apply updated expert knowledge to actual practice

| ○strongly agree |
| --- |
| ○agree |
| ○neutral |
| ○disagree |
| ○strongly disagree |

7.I understand the scope and limits of my team members’ work

| ○strongly agree |
| --- |
| ○agree |
| ○neutral |
| ○disagree |
| ○strongly disagree |

8.I respect my team members’ busy schedules and work pace

| ○strongly agree |
| --- |
| ○agree |
| ○neutral |
| ○disagree |
| ○strongly disagree |

9.I cooperate with my team members to try to solve problems when the team is not functioning well

| ○strongly agree |
| --- |
| ○agree |
| ○neutral |
| ○disagree |
| ○strongly disagree |

10.I reconcile conflicts among team members

| ○strongly agree |
| --- |
| ○agree |
| ○neutral |
| ○disagree |
| ○strongly disagree |

11.I know when problems within the team are likely to arise

| ○strongly agree |
| --- |
| ○agree |
| ○neutral |
| ○disagree |
| ○strongly disagree |

12.I am able to explain the results of my team’s initiative

| ○strongly agree |
| --- |
| ○agree |
| ○neutral |
| ○disagree |
| ○strongly disagree |

13.I am able to adjust my practices to achieve the team’s objectives

| ○strongly agree |
| --- |
| ○agree |
| ○neutral |
| ○disagree |
| ○strongly disagree |

14.I am able to coordinate the opinions of myself and my team members in light of the team’s objective

| ○strongly agree |
| --- |
| ○agree |
| ○neutral |
| ○disagree |
| ○strongly disagree |

15.I provide necessary support to my team members depending on their professional competencies

| ○strongly agree |
| --- |
| ○agree |
| ○neutral |
| ○disagree |
| ○strongly disagree |

16.I am able to objectively evaluate whether the team is operating well

| ○strongly agree |
| --- |
| ○agree |
| ○neutral |
| ○disagree |
| ○strongly disagree |

17.I respect not only the wishes of the patient but also those of the patient’s family

| ○strongly agree |
| --- |
| ○agree |
| ○neutral |
| ○disagree |
| ○strongly disagree |

18.I keep patient independence in mind when providing care

| ○strongly agree |
| --- |
| ○agree |
| ○neutral |
| ○disagree |
| ○strongly disagree |

19.I interact with patients to help them make their own decisions

| ○strongly agree |
| --- |
| ○agree |
| ○neutral |
| ○disagree |
| ○strongly disagree |

20.I change my manner of interacting with patients based on their characteristics and situations

| ○strongly agree |
| --- |
| ○agree |
| ○neutral |
| ○disagree |
| ○strongly disagree |

21.I seek the best way to care for patients

| ○strongly agree |
| --- |
| ○agree |
| ○neutral |
| ○disagree |
| ○strongly disagree |

22.I consciously create opportunities for communication with other professionals

| ○strongly agree |
| --- |
| ○agree |
| ○neutral |
| ○disagree |
| ○strongly disagree |

23.I discuss ideal patient care with other professionals daily

| ○strongly agree |
| --- |
| ○agree |
| ○neutral |
| ○disagree |
| ○strongly disagree |

24.I try to create a suitable atmosphere during meetings wherein it is easy for other professionals to speak

| ○strongly agree |
| --- |
| ○agree |
| ○neutral |
| ○disagree |
| ○strongly disagree |

25.I strive daily to create good interpersonal relationships between professionals

| ○strongly agree |
| --- |
| ○agree |
| ○neutral |
| ○disagree |
| ○strongly disagree |

26.I am able to express opinions in front of other professionals based on my expert knowledge

| ○strongly agree |
| --- |
| ○agree |
| ○neutral |
| ○disagree |
| ○strongly disagree |

27.I fulfill my professional role as required by my team

| ○strongly agree |
| --- |
| ○agree |
| ○neutral |
| ○disagree |
| ○strongly disagree |

28.I understand the scope of what can be accomplished through professional expertise and skills

| ○strongly agree |
| --- |
| ○agree |
| ○neutral |
| ○disagree |
| ○strongly disagree |

29.I am able to state my opinions when necessary from the viewpoint of my professional expertise, even if doing so creates friction with other professionals

| ○strongly agree |
| --- |
| ○agree |
| ○neutral |
| ○disagree |
| ○strongly disagree |

**Part 3 Readiness for Inter-professional Learning Scale (RIPLS)**

**Please select the appropriate option from the following items.**

1.Learning with other students will help me become a more effective member of a health care team

| ○strongly agree |
| --- |
| ○agree |
| ○neutral |
| ○disagree |
| ○strongly disagree |

2.Patients would ultimately benefit if health-care students worked together to solve patient problems

| ○strongly agree |
| --- |
| ○agree |
| ○neutral |
| ○disagree |
| ○strongly disagree |

3.Shared learning with other health-care students will increase my ability to understand clinical problems

| ○strongly agree |
| --- |
| ○agree |
| ○neutral |
| ○disagree |
| ○strongly disagree |

4.Learning with health-care students before qualification would improve relationships after qualification

| ○strongly agree |
| --- |
| ○agree |
| ○neutral |
| ○disagree |
| ○strongly disagree |

5.Communication skills should be learned with other health-care students

| ○strongly agree |
| --- |
| ○agree |
| ○neutral |
| ○disagree |
| ○strongly disagree |

6.Shared learning will help me to think positively about other professionals

| ○strongly agree |
| --- |
| ○agree |
| ○neutral |
| ○disagree |
| ○strongly disagree |

7.For small group learning to work, students need to trust and respect each other

| ○strongly agree |
| --- |
| ○agree |
| ○neutral |
| ○disagree |
| ○strongly disagree |

8.Team-working skills are essential for all health care students to learn

| ○strongly agree |
| --- |
| ○agree |
| ○neutral |
| ○disagree |
| ○strongly disagree |

9.Shared learning will help me to understand my own limitations

| ○strongly agree |
| --- |
| ○agree |
| ○neutral |
| ○disagree |
| ○strongly disagree |

10.I don’t want to waste my time learning with other health-care students

| ○strongly agree |
| --- |
| ○agree |
| ○neutral |
| ○disagree |
| ○strongly disagree |

11.It is not necessary for undergraduate health-care students to learn together

| ○strongly agree |
| --- |
| ○agree |
| ○neutral |
| ○disagree |
| ○strongly disagree |

12.Clinical problem-solving skills can only be learned with students from my own department

| ○strongly agree |
| --- |
| ○agree |
| ○neutral |
| ○disagree |
| ○strongly disagree |

13.Shared learning with other health-care students will help me to communicate better with patients and other professionals

| ○strongly agree |
| --- |
| ○agree |
| ○neutral |
| ○disagree |
| ○strongly disagree |

14.I would welcome the opportunity to work on small-group projects with other health-care students

| ○strongly agree |
| --- |
| ○agree |
| ○neutral |
| ○disagree |
| ○strongly disagree |

15.Shared learning will help to clarify the nature of patient problems

| ○strongly agree |
| --- |
| ○agree |
| ○neutral |
| ○disagree |
| ○strongly disagree |

16.Shared learning before qualification will help me become a better team worker

| ○strongly agree |
| --- |
| ○agree |
| ○neutral |
| ○disagree |
| ○strongly disagree |

17.The function of nurses and therapists is mainly to provide support for doctors

| ○strongly agree |
| --- |
| ○agree |
| ○neutral |
| ○disagree |
| ○strongly disagree |

18.I’m not sure what my professional role will be

| ○strongly agree |
| --- |
| ○agree |
| ○neutral |
| ○disagree |
| ○strongly disagree |

19.I have to acquire much more knowledge and skills than other health-care

students

| ○strongly agree |
| --- |
| ○agree |
| ○neutral |
| ○disagree |
| ○strongly disagree |

**Part 4 Nursing work environment scale (NWES)**

**Please select the appropriate option from the following items.**

1.The hospital supports me in attending in-service education

| ○ strongly agree ○ agree ○ somewhat agree ○ somewhat disagree ○ disagree ○ strongly disagree |
| --- |

2.The hospital can provide me with the necessary reference books and Internet resources

| ○ strongly agree ○ agree ○ somewhat agree ○ somewhat disagree ○ disagree ○ strongly disagree |
| --- |

3.The hospital can provide me with a personalized career pathway

| ○ strongly agree ○ agree ○ somewhat agree ○ somewhat disagree ○ disagree ○ strongly disagree |
| --- |

4.The hospital can provide me with guidance on career planning and encourage me to set learning goals and make career plans

| ○strongly agree |
| --- |
| ○agree  ○somewhat agree |
| ○somewhat disagree |
| ○disagree |
| ○strongly disagree |
|  |

5.The hospital can provide me with a comprehensive pre-job training and mentoring program

| ○strongly agree |
| --- |
| ○agree  ○somewhat agree |
| ○somewhat disagree |
| ○disagree |
| ○strongly disagree |
|  |

6.The leader adopts my suggestions on decision-making

| ○strongly agree |
| --- |
| ○agree  ○somewhat agree |
| ○somewhat disagree |
| ○disagree |
| ○strongly disagree |
| ○strongly agree |

7.When mistakes occur, my leader encourages me to learn from them instead of blaming me

| ○strongly agree |
| --- |
| ○agree  ○somewhat agree |
| ○somewhat disagree |
| ○disagree |
| ○strongly disagree |
|  |

8.The leader is a good listener and give appropriate feedback to the issues I care about

| ○strongly |
| --- |
| ○agree |
| ○somewhat agree |
| ○somewhat disagree |
| ○disagree |
| ○strongly disagree |

9.The leader praises my ideas appropriately and at the right time

| ○strongly |
| --- |
| ○agree |
| ○somewhat agree |
| ○somewhat disagree |
| ○disagree |
| ○strongly disagree |

10.Physicians respect my observations and judgments

| ○strongly |
| --- |
| ○agree |
| ○somewhat agree |
| ○somewhat disagree |
| ○disagree |
| ○strongly disagree |

11.Physicians respect me as a professional

| ○strongly |
| --- |
| ○agree |
| ○somewhat agree |
| ○somewhat disagree |
| ○disagree |
| ○strongly disagree |

12.Physicians recognize my contribution with regard to patients’ recovery

| ○strongly |
| --- |
| ○agree |
| ○somewhat agree |
| ○somewhat disagree |
| ○disagree |
| ○strongly disagree |

13.The relationship between me and physicians is harmonious

| ○strongly |
| --- |
| ○agree |
| ○somewhat agree |
| ○somewhat disagree |
| ○disagree |
| ○strongly disagree |

14.Patients and their relatives recognize my contribution

| ○strongly |
| --- |
| ○agree |
| ○somewhat agree |
| ○somewhat disagree |
| ○disagree |
| ○strongly disagree |

15.Healthcare workers from other professions show recognition of my work

| ○strongly |
| --- |
| ○agree |
| ○somewhat agree |
| ○somewhat disagree |
| ○disagree |
| ○strongly disagree |

16.**I believe my job is valuable**

| ○strongly |
| --- |
| ○agree |
| ○somewhat agree |
| ○somewhat disagree |
| ○disagree |
| ○strongly disagree |

17.I can independently make decisions about care of patients who are in my duty

| ○strongly |
| --- |
| ○agree |
| ○somewhat agree |
| ○somewhat disagree |
| ○disagree |
| ○strongly disagree |

18.I have the freedom to make personalized care measures about my patients

| ○strongly |
| --- |
| ○agree |
| ○somewhat agree |
| ○somewhat disagree |
| ○disagree |
| ○strongly disagree |

19.I have the opportunity to participate in quality improvement projects

| ○strongly |
| --- |
| ○agree |
| ○somewhat agree |
| ○somewhat disagree |
| ○disagree |
| ○strongly disagree |

20.When encountering problems, **I first rely on my own abilities to resolve them**

| ○strongly |
| --- |
| ○agree |
| ○somewhat agree |
| ○somewhat disagree |
| ○disagree |
| ○strongly disagree |

21.I am satisfied with the benefits

| ○strongly |
| --- |
| ○agree |
| ○somewhat agree |
| ○somewhat disagree |
| ○disagree |
| ○strongly disagree |

22.I am satisfied with my vacation

| ○strongly |
| --- |
| ○agree |
| ○somewhat agree |
| ○somewhat disagree |
| ○disagree |
| ○strongly disagree |

23.I am satisfied with my salary

| ○strongly |
| --- |
| ○agree |
| ○somewhat agree |
| ○somewhat disagree |
| ○disagree |
| ○strongly disagree |

24.I have enough time to communicate with my patients

| ○strongly |
| --- |
| ○agree |
| ○somewhat agree |
| ○somewhat disagree |
| ○disagree |
| ○strongly disagree |

25.I have enough time and opportunities to discuss patient care problems with my colleagues

| ○strongly |
| --- |
| ○agree |
| ○somewhat agree |
| ○somewhat disagree |
| ○disagree |
| ○strongly disagree |

26.The nursing staffing of my unit meets its needs

| ○strongly |
| --- |
| ○agree |
| ○somewhat agree |
| ○somewhat disagree |
| ○disagree |
| ○strongly disagree |
