# Supplementary material for: Interprofessional collaboration and associated factors among wound, ostomy, and continence nurses: a cross-sectional study in China
Source: PeerJ. 2025 Sep 30;13:e20006. doi: 10.7717/peerj.20006 (PMC12493770; doi:10.7717/peerj.20006)
Supplement: Supplemental Information 5 [file peerj-13-20006-s005.docx]

**伤口造口专科护士跨专业协作能力调查问卷**

**第一部分 一般资料**

1.您的性别：

| ○男 | ○女 |  |  |  |  |  |  |
| --- | --- | --- | --- | --- | --- | --- | --- |

2.年龄（周岁）：_________________________________

3.您是否为独生子女？

| ○是 |
| --- |
| ○否 |

4.您的婚姻状况：

| ○未婚 |
| --- |
| ○已婚 |
| ○离异 |

5.您从事伤口造口专科护士的工作年限（年）： _________________________________

6.您的职称：

| ○初级 |
| --- |
| ○中级 |
| ○高级 |

7.您的职务

| ○护士 |
| --- |
| ○护士长 |

8.您的最高学历：

| ○专科 |
| --- |
| ○本科 |
| ○硕士研究生  ○博士研究生 |

9.医院级别：

| ○三级甲等 |
| --- |
| ○三级乙等 |
| ○二级甲等 |

10.近3年内您是否有院外交流经历？

| ○无 |
| --- |
| ○有 |

11.您的用工方式：

| ○编制 |
| --- |
| ○合同 |

12.您的专科工作形式：

| ○专职 |
| --- |
| ○兼职 |

13.您是否担任伤口造口专科组长？

| ○是 |
| --- |
| ○否 |

**第二部分**在你与其他专业人员协作时，你是如何理解协作并实施协作的？在如下条目中选择您认为合适的选项。**本量表的“团队”是指以患者的治疗、照护为目的的多专业人员所构成的团队（如伤口造口专科护士和不同学科的医生等专业人员组成的团队）**

1.一直在努力改善实践

| ○完全同意 |
| --- |
| ○基本同意 |
| ○中立 |
| ○不同意 |
| ○完全不同意 |

2.经常反思已实施的治疗和护理措施

| ○完全同意 |
| --- |
| ○基本同意 |
| ○中立 |
| ○不同意 |
| ○完全不同意 |

3.追求专业人员应具备的专业素养

| ○完全同意 |
| --- |
| ○基本同意 |
| ○中立 |
| ○不同意 |
| ○完全不同意 |

4.实施有依据的治疗、护理

| ○完全同意 |
| --- |
| ○基本同意 |
| ○中立 |
| ○不同意 |
| ○完全不同意 |

5.对任何人做任何治疗、护理都能说明其依据

| ○完全同意 |
| --- |
| ○基本同意 |
| ○中立 |
| ○不同意 |
| ○完全不同意 |

6.能在实践中灵活应用最新的专业知识

| ○完全同意 |
| --- |
| ○基本同意 |
| ○中立 |
| ○不同意 |
| ○完全不同意 |

7.理解团队成员的工作范围和界限

| ○完全同意 |
| --- |
| ○基本同意 |
| ○中立 |
| ○不同意 |
| ○完全不同意 |

8.能考虑到团队成员的忙碌程度和工作节奏

| ○完全同意 |
| --- |
| ○基本同意 |
| ○中立 |
| ○不同意 |
| ○完全不同意 |

9.团队工作不顺利时，团队成员能够尝试协作解决问题

| ○完全同意 |
| --- |
| ○基本同意 |
| ○中立 |
| ○不同意 |
| ○完全不同意 |

10.团队成员之间出现分歧时能够自行调整

| ○完全同意 |
| --- |
| ○基本同意 |
| ○中立 |
| ○不同意 |
| ○完全不同意 |

11.知道什么时候团队最易出现分歧

| ○完全同意 |
| --- |
| ○基本同意 |
| ○中立 |
| ○不同意 |
| ○完全不同意 |

12.能说明团队合作取得的成果

| ○完全同意 |
| --- |
| ○基本同意 |
| ○中立 |
| ○不同意 |
| ○完全不同意 |

13.能为团队能达成目标而调整自己的实践活动

| ○完全同意 |
| --- |
| ○基本同意 |
| ○中立 |
| ○不同意 |
| ○完全不同意 |

14.自己根据团队的目标协调与团队成员之间的意见

| ○完全同意 |
| --- |
| ○基本同意 |
| ○中立 |
| ○不同意 |
| ○完全不同意 |

15.能为团队成员提供必要的专业性支持

| ○完全同意 |
| --- |
| ○基本同意 |
| ○中立 |
| ○不同意 |
| ○完全不同意 |

16.能客观评价团队工作是否顺利进行

| ○完全同意 |
| --- |
| ○基本同意 |
| ○中立 |
| ○不同意 |
| ○完全不同意 |

17.尊重患者和家属的意见

| ○完全同意 |
| --- |
| ○基本同意 |
| ○中立 |
| ○不同意 |
| ○完全不同意 |

18.在治疗、护理中鼓励患者自理

| ○完全同意 |
| --- |
| ○基本同意 |
| ○中立 |
| ○不同意 |
| ○完全不同意 |

19.在采取任何护理活动时，能充分考虑患者的真实意愿

| ○完全同意 |
| --- |
| ○基本同意 |
| ○中立 |
| ○不同意 |
| ○完全不同意 |

20.根据患者的特点和情况改变治疗、护理方案

| ○完全同意 |
| --- |
| ○基本同意 |
| ○中立 |
| ○不同意 |
| ○完全不同意 |

21.探究对患者最有利的治疗及护理措施

| ○完全同意 |
| --- |
| ○基本同意 |
| ○中立 |
| ○不同意 |
| ○完全不同意 |

22.有意识地创造与其他专业人员交流的机会

| ○完全同意 |
| --- |
| ○基本同意 |
| ○中立 |
| ○不同意 |
| ○完全不同意 |

23.经常与其他专业人员交流患者的治疗及护理情况

| ○完全同意 |
| --- |
| ○基本同意 |
| ○中立 |
| ○不同意 |
| ○完全不同意 |

24.开会时尽量营造一种与其他专业人员方便交流的氛围

| ○完全同意 |
| --- |
| ○基本同意 |
| ○中立 |
| ○不同意 |
| ○完全不同意 |

25.平时注意努力与其他专业人员建立良好的人际关系

| ○完全同意 |
| --- |
| ○基本同意 |
| ○中立 |
| ○不同意 |
| ○完全不同意 |

26.能根据专业知识向其他专业人员提供建议

| ○完全同意 |
| --- |
| ○基本同意 |
| ○中立 |
| ○不同意 |
| ○完全不同意 |

27.能够按照团队要求提供自己的专业技术

| ○完全同意 |
| --- |
| ○基本同意 |
| ○中立 |
| ○不同意 |
| ○完全不同意 |

28.理解自己专业领域的知识和技术的范围

| ○完全同意 |
| --- |
| ○基本同意 |
| ○中立 |
| ○不同意 |
| ○完全不同意 |

29.即使与其他专业人员意见有冲突，也能从自身专业角度提出必要的意见

| ○完全同意 |
| --- |
| ○基本同意 |
| ○中立 |
| ○不同意 |
| ○完全不同意 |

**第三部分**请您在如下条目中选择您认为合适的选项。

1.与其他专业人员一起学习有助于我成为更有效的健康照护团队中一员

| ○非常同意 |
| --- |
| ○同意 |
| ○中立 |
| ○不同意 |
| ○非常不同意 |

2.如果健康照护团队专业人员共同工作解决患者的问题，那么患者终将受益

| ○非常同意 |
| --- |
| ○同意 |
| ○中立 |
| ○不同意 |
| ○非常不同意 |

3.与其他健康照护团队专业人员共享学习将会提高我理解临床问题的能力

| ○非常同意 |
| --- |
| ○同意 |
| ○中立 |
| ○不同意 |
| ○非常不同意 |

4.在获得（专业）资质前，与健康照护（专业）学生一起学习能够增进团队关系

| ○非常同意 |
| --- |
| ○同意 |
| ○中立 |
| ○不同意 |
| ○非常不同意 |

5.沟通技能应与其他健康照护（专业）的学生一起学习

| ○非常同意 |
| --- |
| ○同意 |
| ○中立 |
| ○不同意 |
| ○非常不同意 |

6.共享学习将帮助我积极思考其他专业

| ○非常同意 |
| --- |
| ○同意 |
| ○中立 |
| ○不同意 |
| ○非常不同意 |

7.在小组学习中，学生需要彼此信任和尊重

| ○非常同意 |
| --- |
| ○同意 |
| ○中立 |
| ○不同意 |
| ○非常不同意 |

8.团队合作的技能对所有健康照护（专业）的学生都是必不可少的

| ○非常同意 |
| --- |
| ○同意 |
| ○中立 |
| ○不同意 |
| ○非常不同意 |

9.共享学习将帮助我了解自身的不足

| ○非常同意 |
| --- |
| ○同意 |
| ○中立 |
| ○不同意 |
| ○非常不同意 |

10.我不想浪费时间与其他健康照护（专业）的学生一起学习

| ○非常同意 |
| --- |
| ○同意 |
| ○中立 |
| ○不同意 |
| ○非常不同意 |

11.健康照护（专业）的本科学生没有必要在一起学习

| ○非常同意 |
| --- |
| ○同意 |
| ○中立 |
| ○不同意 |
| ○非常不同意 |

12.临床问题解决技能只有从与我同系的同学一起学习获得

| ○非常同意 |
| --- |
| ○同意 |
| ○中立 |
| ○不同意 |
| ○非常不同意 |

13.与其他健康照护（专业）的学生共享学习将帮助我与患者和其他专业人士更好地沟通

| ○非常同意 |
| --- |
| ○同意 |
| ○中立 |
| ○不同意 |
| ○非常不同意 |

14.我十分乐意接受与其他健康照护（专业）的学生进行小组项目合作的机会

| ○非常同意 |
| --- |
| ○同意 |
| ○中立 |
| ○不同意 |
| ○非常不同意 |

15.共享学习将有助于明确患者问题的实质

| ○非常同意 |
| --- |
| ○同意 |
| ○中立 |
| ○不同意 |
| ○非常不同意 |

16.在获得（专业）资质前，共享学习将帮助我成为一名更好的团队成员

| ○非常同意 |
| --- |
| ○同意 |
| ○中立 |
| ○不同意 |
| ○非常不同意 |

17.护士和治疗师的主要功能是为医生提供支持

| ○非常同意 |
| --- |
| ○同意 |
| ○中立 |
| ○不同意 |
| ○非常不同意 |

18.我不确定我的专业角色将会是什么

| ○非常同意 |
| --- |
| ○同意 |
| ○中立 |
| ○不同意 |
| ○非常不同意 |

19.我必须比其他健康照护（专业）的学生获得更多的知识和技能

| ○非常同意 |
| --- |
| ○同意 |
| ○中立 |
| ○不同意 |
| ○非常不同意 |

**第四部分**请您在如下条目中选择您认为合适的选项。

1.医院支持我进行在职教育

| ○非常同意 |
| --- |
| ○同意 |
| ○有点同意 |
| ○有点不同意 |
| ○不同意 |
| ○非常不同意 |

2.医院能为我提供所需的参考书籍和网络资源

| ○非常同意 |
| --- |
| ○同意 |
| ○有点同意 |
| ○有点不同意 |
| ○不同意 |
| ○非常不同意 |

3.医院能为我提供个性化的发展空间

| ○非常同意 |
| --- |
| ○同意 |
| ○有点同意 |
| ○有点不同意 |
| ○不同意 |
| ○非常不同意 |

4.医院能为我进行职业规划指导，鼓励我设定学习目标和职业规划

| ○非常同意 |
| --- |
| ○同意 |
| ○有点同意 |
| ○有点不同意 |
| ○不同意 |
| ○非常不同意 |

5.医院能为我提供完善的岗前培训和带教计划

| ○非常同意 |
| --- |
| ○同意 |
| ○有点同意 |
| ○有点不同意 |
| ○不同意 |
| ○非常不同意 |

6.在决策上，领导会听取我的建议

| ○非常同意 |
| --- |
| ○同意 |
| ○有点同意 |
| ○有点不同意 |
| ○不同意 |
| ○非常不同意 |

7.出现差错时，领导鼓励我吸取教训而非谴责

| ○非常同意 |
| --- |
| ○同意 |
| ○有点同意 |
| ○有点不同意 |
| ○不同意 |
| ○非常不同意 |

8.领导善于倾听，并能对我所关心的问题给予相应答复

| ○非常同意 |
| --- |
| ○同意 |
| ○有点同意 |
| ○有点不同意 |
| ○不同意 |
| ○非常不同意 |

9.领导会适时适当称赞我的想法

| ○非常同意 |
| --- |
| ○同意 |
| ○有点同意 |
| ○有点不同意 |
| ○不同意 |
| ○非常不同意 |

10.医生尊重我的观察和判断

| ○非常同意 |
| --- |
| ○同意 |
| ○有点同意 |
| ○有点不同意 |
| ○不同意 |
| ○非常不同意 |

11.医生尊重我的专业性

| ○非常同意 |
| --- |
| ○同意 |
| ○有点同意 |
| ○有点不同意 |
| ○不同意 |
| ○非常不同意 |

12.医生认可我在患者康复中所做的贡献

| ○非常同意 |
| --- |
| ○同意 |
| ○有点同意 |
| ○有点不同意 |
| ○不同意 |
| ○非常不同意 |

13.我和医生之间的关系融洽

| ○非常同意 |
| --- |
| ○同意 |
| ○有点同意 |
| ○有点不同意 |
| ○不同意 |
| ○非常不同意 |

14.我在工作中受到患者及其家属的肯定

| ○非常同意 |
| --- |
| ○同意 |
| ○有点同意 |
| ○有点不同意 |
| ○不同意 |
| ○非常不同意 |

15.我在工作中受到其他医务人员的认可

| ○非常同意 |
| --- |
| ○同意 |
| ○有点同意 |
| ○有点不同意 |
| ○不同意 |
| ○非常不同意 |

16.我觉得自己的工作是有价值的

| ○非常同意 |
| --- |
| ○同意 |
| ○有点同意 |
| ○有点不同意 |
| ○不同意 |
| ○非常不同意 |

17.我能独立决定患者的照护及工作上的问题

| ○非常同意 |
| --- |
| ○同意 |
| ○有点同意 |
| ○有点不同意 |
| ○不同意 |
| ○非常不同意 |

18.我有权对自己负责的患者提出个性化的护理措施

| ○非常同意 |
| --- |
| ○同意 |
| ○有点同意 |
| ○有点不同意 |
| ○不同意 |
| ○非常不同意 |

19.我有机会参与质量改进项目

| ○非常同意 |
| --- |
| ○同意 |
| ○有点同意 |
| ○有点不同意 |
| ○不同意 |
| ○非常不同意 |

20.遇到问题时，我首先依靠自己的能力来解决

| ○非常同意 |
| --- |
| ○同意 |
| ○有点同意 |
| ○有点不同意 |
| ○不同意 |
| ○非常不同意 |

21.我对所获得的福利感到满意

| ○非常同意 |
| --- |
| ○同意 |
| ○有点同意 |
| ○有点不同意 |
| ○不同意 |
| ○非常不同意 |

22.我对所获得的休假时间感到满意

| ○非常同意 |
| --- |
| ○同意 |
| ○有点同意 |
| ○有点不同意 |
| ○不同意 |
| ○非常不同意 |

23.我对所获得的薪资报酬感到满意

| ○非常同意 |
| --- |
| ○同意 |
| ○有点同意 |
| ○有点不同意 |
| ○不同意 |
| ○非常不同意 |

24.我有足够的时间与患者沟通交流

| ○非常同意 |
| --- |
| ○同意 |
| ○有点同意 |
| ○有点不同意 |
| ○不同意 |
| ○非常不同意 |

25.我有足够的时间和机会与其他护士讨论患者的护理问题

| ○非常同意 |
| --- |
| ○同意 |
| ○有点同意 |
| ○有点不同意 |
| ○不同意 |
| ○非常不同意 |

26.科室的护理人力资源配置能满足临床的需求

| ○非常同意 |
| --- |
| ○同意 |
| ○有点同意 |
| ○有点不同意 |
| ○不同意 |
| ○非常不同意 |
