# Supplementary material for: Interprofessional collaboration and associated factors among wound, ostomy, and continence nurses: a cross-sectional study in China
Source: PeerJ. 2025 Sep 30;13:e20006. doi: 10.7717/peerj.20006 (PMC12493770; doi:10.7717/peerj.20006)
Supplement: Supplemental Information 2 [file peerj-13-20006-s002.docx]

**Dear editor：**

Here is the English translation for Sheet 3 from the raw data. I also sincerely apologize for providing an incorrect response previously.

Off-site learning experience（院外交流） no（无）=1 yes（有）=2

Administrative position（职务） nurse（护士）=1 nursing management （护士长）=2

Serving as a specialty team leader（组长） yes（是）=1 no（否）=2
